# Agent-Based Deterministic Modeling of the Bone Marrow Homeostasis

**DOI:** 10.1155/2016/8054219

**Published:** 2016-06-02

**Authors:** Manish Kurhekar, Umesh Deshpande

**Affiliations:** Department of Computer Science and Engineering, VNIT Nagpur, Nagpur 440010, India

## Abstract

Modeling of stem cells not only describes but also predicts how a stem cell's environment can control its fate. The first stem cell populations discovered were hematopoietic stem cells (HSCs). In this paper, we present a deterministic model of bone marrow (that hosts HSCs) that is consistent with several of the qualitative biological observations. This model incorporates stem cell death (apoptosis) after a certain number of cell divisions and also demonstrates that a single HSC can potentially populate the entire bone marrow. It also demonstrates that there is a production of sufficient number of differentiated cells (RBCs, WBCs, etc.). We prove that our model of bone marrow is biologically consistent and it overcomes the biological feasibility limitations of previously reported models. The major contribution of our model is the flexibility it allows in choosing model parameters which permits several different simulations to be carried out in silico without affecting the homeostatic properties of the model. We have also performed agent-based simulation of the model of bone marrow system proposed in this paper. We have also included parameter details and the results obtained from the simulation. The program of the agent-based simulation of the proposed model is made available on a publicly accessible website.

## 1. Introduction

Stem cells and their descendants are the building blocks of life. How stem cell populations guarantee their maintenance and self-renewal and how individual stem cells decide to transit from one cell state to another to generate mature differentiated cells are long standing and fascinating questions [1]. There is a significant interest in studying stem cells, both to elucidate their basic biological functions and to learn how to utilize them as new sources of specialized cells for tissue repair [2].

Blood is the life preserving fluid, whose major functions are supply of nutrients and oxygen to the tissues, self-immunity, and defense against pathogens. In order to carry out these tasks, human blood contains a variety of cells, each precisely adapted to its specific objective. All the different blood cells develop from a kind of master cell, called the hematopoietic (blood forming) stem cell (HSC). Incidentally, the first stem cell populations discovered were HSCs. HSCs are primarily present in the bone marrow. HSCs are stem cells that give rise to all the differentiated blood cell types including white blood cells (WBC), red blood cells (RBCs), and platelets. Fully mature differentiated cells migrate into the blood stream. The transition of HSCs from quiescence (not undergoing any cell cycle) into proliferation, or differentiation, is governed by their internal state and by chemicals secreted by neighboring cells in their immediate microenvironment.

It is believed that a single HSC is sufficient to reconstitute the entire blood system [3, 4]. This extraordinary regenerative ability of the bone marrow is not surprising, considering that it has a vital role that must remain unaffected by stem cells depletion that might occur, for example, as a result of chemotherapy, radiation, or disease. It should be emphasized that though the supply of blood cells in the periphery is steady, the bone marrow is not static. It is dynamic in the sense that it constantly changes in its constitution and arrangement, and these changes occur at varying rates. The bone marrow is in a state of homeostasis that can be considered as a dynamic equilibrium between its constituents.

Theise and Harris [5] describe how stem cells and their lineages are examples of complex adaptive systems. Profound understanding of a complex adaptive system can be gathered by generating computer models using computational techniques. Agent-based modeling is a way to represent such complex adaptive systems in software. An agent is a high-level software abstraction that provides a convenient and powerful way to describe a complex software entity in terms of its behavior within a contextual computational environment. Agents are flexible problem-solving computational entities that are reactive (respond to the environment) and autonomous (not externally controlled) and interact with other such entities.

One of the significant contributions to stem cell modeling was by Agur et al. [6]. The main aim of their paper was to provide a mathematical basis for the bone marrow homeostasis. More precisely, they wanted to define the properties that enabled the bone marrow to rapidly return to a steady supply of blood cells after relatively large perturbations in stem cell numbers. Their model is represented as a family of cellular automata on a connected, locally finite undirected graph. Their model can be briefly described as follows: It has three types of cells, stem cells, differentiated cells, and null cells. Each cell has an internal counter. Stem cells differentiate when their immediate neighborhood is saturated with stem cells and their internal counter reaches a certain threshold. A differentiated cell converts to a null cell after its internal counter crosses the required threshold, a process that denotes the passing of a differentiated cell to blood stream leaving the place it had earlier occupied in the bone marrow empty. A null cell, with a stem cell neighbor, is converted to a stem cell when its internal counter reaches a particular threshold.

D'Inverno and Saunders [7] have listed the following drawbacks of Agur et al.'s [6] model:

1. The specification of Agur et al.'s model reveals that the null cells must have counters. In a sense, an empty space has to do some computational work. This lacks biological feasibility and is against what the paper states about modeling cells having counters, rather than empty locations.
2. Stem cell division is not explicitly represented; instead, stem cells are brought into existence in empty spaces.
3. A stem cell appears when a null cell has been surrounded by at least one stem cell for a particular period. However, the location of the neighboring stem cell can vary at each step.
4. As an effect of the drawback mentioned above, a stem cell can potentially differentiate more than once in the same time instant since it might be surrounded by more than one null cell. Hence, potentially more than one neighboring null cells can get converted to stem cells.
5. The state of a stem cell after division is not defined. Nothing is said about what happens to a stem cell after a new stem cell appears in the null cell space. There is no provision of any preconditions on the stem cell division.
6. There is no provision for stem cell apoptosis.

D'Inverno and Saunders [7] provided an agent-based simulation for the model described by Agur et al. They needed to overcome the third limitation mentioned above for creating a deterministic agent-based simulation. In order to overcome the limitation, they introduced the concept of a controlling microenvironment that links a null cell, which has reached a threshold, with a stem cell that can differentiate. All the cells send and receive signals from the microenvironment and act on its suggestions. They performed agent-based implementation with the incorporation of Agur et al.'s model in two dimensions. However, the improvement suggested by them, of a controlling microenvironment, does not have any biological basis.

Moreover, there is an additional limitation of the model described by Agur et al. The limitation is that there are no intermediate cells, also called transitive cells, in the model proposed by them. Transitive cells have limited stem cell-like properties that decreases with each subsequent generation and they are eventually converted to differentiated cells. For hematopoietic system, common lymphoid progenitor (CLP) and common myeloid progenitor (CMP) are examples of transitive cells [8]. As there are no transitive cells, there cannot be any conversion of a transitive cell to a stem cell, which can help bone marrow system to recover in case of severe perturbations.

Some other novel models of HSCs are proposed. Roeder and Loeffler [9] propose a stochastic model with two growth environments where a stem cell remains quiescent for longer periods of time when it is in first environment and proliferates when it transitions to the other environment. The proliferation and transition depend only upon two stochastic parameters. Glauche et al. [10] provide two independent compartments for fast proliferating HSCs and slow proliferating HSCs to explain simultaneous occurrence of self-renewal and differentiation. Glauche et al. [11] further improved their model by considering the effects of aging on stem cell population. These models show within-tissue plasticity and proliferation and self-renewal potential of stem cells. Stem cells moving between two different compartments or environments with a fixed probability are an artifact that is not biologically consistent. The probability might change since it is dependent on the local environment of the stem cell. Another limitation is that these models show homeostasis for only a limited range of parameter values.

The model proposed in this paper is an enhancement over our earlier model [12], with incorporation of stem cell death (apoptosis) after certain number of cell divisions. In our model, we have addressed all the limitations listed above by extending and augmenting the model originally proposed by Agur et al., thereby making the model close to biological observations. The model we present is aimed at simulating a situation in which a cell's behavior is determined only by a combination of the types and states of cells in its proximity and its own cell cycle represented by its internal counters. The main assumptions of our model are as follows:

- Cell behavior is determined by the number and type of its neighbors. This assumption is aimed at describing the fact that cytokines, secreted by cells into the microenvironment, are capable of activating cells into changing their types [1, 3].
- Each cell has an internal counter that determines the time required for it to mature. The duration for maturity is fixed for each type of cell. After maturity, the cell changes its type or its generation.
- Stem cell apoptosis occurs after certain number of renewals.
- Every cell possesses a directional component and it proliferates in the direction of that component. The directional component is updated after each proliferation. Although the directional component has no biological significance, it allows the model to be fully deterministic.
- The model captures emergent behavior of the bone marrow that is consistent with several biological observations:
  (a) The model has high resilience for the bone marrow homeostasis as shown in [13].
  (b) The model incorporates intermediate transitive cells and quiescent stem cells as described in [8].
  (c) In [14], the authors mention that the transitive cells can become stem cells in exceptional circumstances. The model supports this observation.
  (d) The model also incorporates stem cell apoptosis as given in [15].
- The model does not account for leukemia causing abnormal stem cell behaviors as is done in [16].

We have performed agent-based simulation of the model of bone marrow stem cell system proposed in this paper. The details and the results of this simulation are provided in the Appendix.

The paper is organized as follows: In the next section, we describe our model and the rules that govern it. In Section 3, we show how a single stem cell can populate the entire bone marrow and also prove the homeostatic properties of the proposed model. In Section 4, we show that the model provides a steady supply of differentiated cells to the blood stream and we also show that several stem cells remain in quiescent state. In Section 5, we describe the steady states and death states of our proposed model. We discuss the theoretical results in Section 6. The results of the agent-based simulation are included in the Appendix.

## 2. Description of the Model

Our model contains three basic types of cells and a notation for empty space:

(i) *Stem cell*, denoted by *S*, either can proliferate generating new stem cells or can convert to a transitive cell. They can become quiescent. In the event of the death of a stem cell, it can be considered to be converting to an empty space.
(ii) *Transitive cell*, denoted by *T*, either can convert to a differentiated cell or can convert back to a stem cell when there are no stem cells in its near neighborhood.
(iii) *Differentiated cell*, denoted by *D*, is the final product of a stem cell. After maturation, these cells leave the bone marrow.
(iv) *Empty space*, represented by *E*, denotes space in the bone marrow that can be occupied by either a stem cell or a transitive cell or a differentiated cell.

In our model, the bone marrow is represented as a connected, locally finite undirected graph. This describes the neighborhood of bone marrow cells.

**Figure 1:**
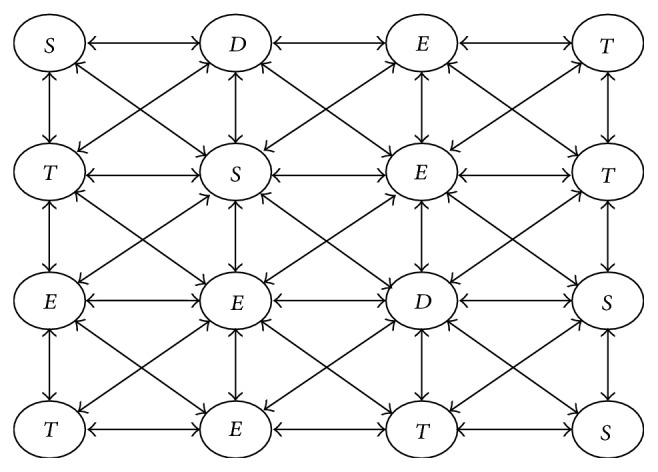
Bone marrow graph in two dimensions (each vertex is a cell that has eight immediate neighbors and the label denotes its type).

Let *G* = (*V*, *L*) be a connected, locally finite undirected graph that denotes the bone marrow. Its vertex set *V* denotes the cells and the set of edges *L* describes the neighboring cells to which a cell is connected in the bone marrow (Figure 1).

**Figure 2:**
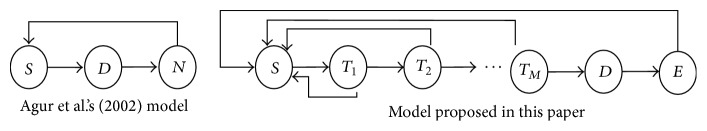
Comparison of Agur et al.'s model and the model proposed in this paper.

Diagrammatically, the transitions of different types of cells in Agur et al.'s [6] model and our proposed model are depicted in Figure 2 (*N* denotes a null cell in Agur et al.'s model).

For every pair of vertices *u*, *v* ∈ *V*, we denote by *ρ*(*u*, *v*) the distance between these vertices in the shortest-path metric induced by *G*. *N*(*v*) = {*u* ∈ *V*∣*ρ*(*u*, *v*) = 1} denotes the* immediate neighborhood* of a vertex *v* ∈ *V*, that is, the set of vertices joined to *v* by an edge. *B*(*v*, *n*) denotes the* ball of radius n* centered in *v* ∈ *V*. It is the set of all vertices such that their distances from *v* do not exceed *n*. We write *B*(*v*, *n*) = {*u* ∈ *V*∣*ρ*(*u*, *v*) ≤ *n*}. *B*(*v*, *n*) defines the* near neighborhood* of size *n* of vertex *v*. If *U*⊆*V* is a nonempty subset of vertices, then for every vertex *v* ∈ *V* let *ρ*_*U*_(*v*) = min_*u*∈*U*_ *ρ*(*u*, *v*) be the minimum distance between *v* and another vertex *u* ∈ *U*.

The* state* of a vertex is a 1-tuple, a 2-tuple, a 3-tuple, or a 4-tuple depending on the cell type. The first coordinate of the tuple denotes the cell's type (*S*, *T*, *D*, or *E* denoting a stem cell, a transitive cell, a differentiated cell, or an empty space, resp.). The state of a stem cell is defined by a 4-tuple. The second coordinate denotes the direction of proliferation, which generally rotates clockwise or counterclockwise. An example will later explain the implication of direction of rotation. The third coordinate denotes the simulated time *τ* as an internal counter. The last coordinate denotes the number of times the stem cell has proliferated. The state of a transitive cell is defined by a 3-tuple. The second coordinate denotes its generation (progeny) while the third coordinate denotes the simulated time *τ*. The state of a differentiated cell is defined by a 2-tuple and the second coordinate denotes the simulated time *τ*. Finally, the state of an empty space has a single coordinate that denotes its type.

Let *μ* be the maximum number of immediate neighbors possible for any cell. Thus, *μ* also denotes the number of directions for a stem cell to proliferate. When it proliferates, a stem cell can occupy an empty space, if available, in its immediate neighborhood. A transitive cell can go through several generations (progeny) before it converts to a differentiated cell. These are several differentiation stages for a transitive cell. A transitive cell moves from one generation to the next after its internal counter reaches a certain threshold. There are *M* generations for a transitive cell, where *M* is greater than or equal to 1. When a transitive cell has moved into its last generation (i.e., the *M*th generation) and when its internal counter reaches a certain threshold, it converts to a differentiated cell. In circumstances when there is not even a single stem cell in the near neighborhood of a transitive cell, a transitive cell converts back to a stem cell (dedifferentiation [5, 17]). Such extreme rare circumstances can be observed in very specific conditions, for example, due to radiation and organ damage. The rules given below also capture the fact that a transitive cell's ability to convert back to a stem cell diminishes with each subsequent generation. The parameter *η* denotes the distance multiple for a transitive cell to convert back to a stem cell. The conversion from a transitive cell to a stem cell depends on the distance multiple *η* and its current generation. To have nonzero near neighborhood for a transitive cell, the generation counter for transitive cells starts with 1.

Let *Ω* be the set of states of a vertex.

A map *x* : *V* → *Ω* is the state of the entire graph. The set of all the states of the bone marrow graph *G* is denoted by *Ω* ^*V*^. A state *x* ∈ *Ω* ^*V*^ of the bone marrow graph *G* at time *t* is denoted by *x* ^*t*^. The state of a vertex *v* at time *t* is denoted by *x* ^*t*^(*v*).

With the above definitions, we are now ready to define the rules of an iterative operator on all states *Ω* ^*V*^. It depends on four* positive nonzero* integers: constant for stem cells maturity Ψ, constant for transitive cells maturity Θ, constant for differentiated cells maturity Φ, and maximum number of stem cells renewals Δ. The rules for the state changes can be regarded as describing a family of cellular automata. We have used ∨ for logical-or and ∧ for logical-and.

There are four rules that describe our model, one for each type of cell. Some rules have subrules. The order of evaluation of subrules is based on the order they are specified in that rule. Hence, at every time instant, for every cell, only one subrule will be applied deterministically. Note that “*∗*” denotes any possible value:
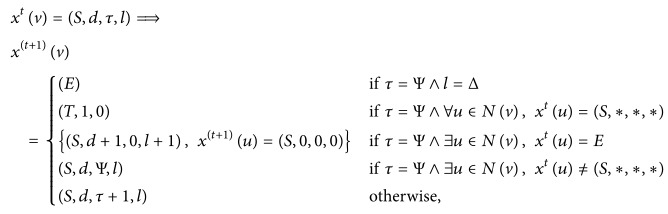
(1)

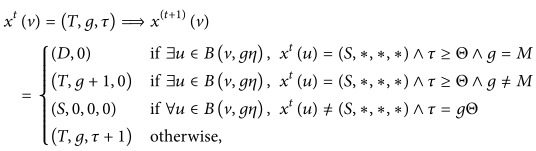
(2)

(3) xtv=D,τ⟹xt+1v=Eif  τ=ΦD,τ+1otherwise,

(4) xtv=E⟹xt+1v=E.

These rules are described in detail below.

Rule (1) is for stem cells. The first subrule of Rule (1) says that every stem cell renews Δ number of times. If the stem cell has renewed itself Δ times, it dies and it is removed from the bone marrow. The space occupied by the stem cell then becomes empty space. Wang et al. [18] provide experimental evidence for a differentiation checkpoint that limits self-renewal of HSCs. The next subrule of Rule (1) states that a stem cell converts to a transitive cell, if its internal counter representing its cycling phase has reached the threshold Ψ and its immediate neighborhood consists only of stem cells. This corresponds to receiving a signal that the microenvironment is saturated with stem cells. de Haan et al. [3] provide the evidence for such a feedback, where the authors show that hematopoietic cell amplification in vivo is regulated by various mechanisms that appear to be under the control of many hematopoietic growth factors, including the activation and deactivation of the quiescent stem cells into the cell cycle. The next subrule states that when a stem cell's internal counter reaches the threshold Ψ and there exists an empty space in its neighborhood, then it proliferates such that one of its descendants occupies the empty space and the other remains in the original location. This subrule also defines the fact that the new stem cell as well as the stem cell at the original location receives renewed biological time. With these subrules, we also denote a systematic way of choosing the empty space for proliferation. The method we propose is by adding a directional component *d* in the state of every stem cell and by arranging all the possible directions *μ* in a circular (round-robin) order (in two-dimensional or three-dimensional model of space). A stem cell proliferates in the empty space in the directional component *d* of its state. If the position given by the directional component *d* of the state is occupied by any cell, then the stem cell continues to choose the next direction, in the round-robin order, for availability of the empty space. After proliferation, the directional component of stem cell is incremented to point to the next subsequent direction. The fourth subrule within this rule specifies that if a stem cell's internal counter has reached the threshold Ψ but its immediate neighborhood is not saturated by stem cells and it also does not have empty space to proliferate (as earlier subrules are already considered), then the stem cell enters a quiescent state; that is, it retains its state. The last subrule states that if the internal counter of a stem cell has not reached the threshold Ψ, then it is incremented.

Rule (2) details the behavior of transitive cells. The first and second subrules state that when a transitive cell's internal counter reaches the threshold Θ and if there exists a stem cell in its near neighborhood, then it moves onto the next generation unless it is not in its last (*M*th) generation. If a transitive cell's counter has reached the threshold Θ and it is in its last generation, then it gets converted to a differentiated cell. Transitive cells are intermediate cells that can convert back to stem cells if there are no enough stem cells in their near neighborhood, a situation that can occur following radiation or organ damage. Theise and Harris [5] detail the dedifferentiation, that is, reversion of an intermediate transitive cell into a stem cell. The near neighborhood of a transitive cell is governed by a constant *η* and the generation of the transitive cell. This is based on the observation that the* stemness* property of a transitive cell goes on decreasing with subsequent generations. The near neighborhood size to find a stem cell and the duration *g*Θ required for maturity keep on increasing with each subsequent generation of a transitive cell, implying its reduced capacity to regenerate and the requirement of an even stronger signal to convert back to a stem cell. The third subrule captures this behavior. The last subrule states that if the internal counter of a transitive cell has not reached the threshold Θ, then it is incremented.

In Rule (3), the first subrule states that when a differentiated cell's internal counter reaches the threshold Φ, it matures. After maturation, the cell migrates to the blood stream leaving the original space occupied by the differentiated cell as empty space. The second subrule states that if the internal counter has not reached the threshold Φ, then it is incremented.

Rule (4) specifies that an empty space does not change by itself. It does not have any internal counter and it is not involved in any computation.

The rules defined for this model are complete and consistent. The bone marrow has three types of cells: the stem cells, the transitive cells, and the differentiated cells. The rules, for all the cell types, are defined such that each state of the cell matches some subrule. For example, in Rule (1) for stem cells, if the internal counter of a stem cell reaches the threshold Ψ and if it has not had Δ proliferations, then it either can convert to a transitive cell if it is surrounded by stem cells from all directions or may proliferate into a new stem cell in an empty space in its neighborhood; or if neither of this is possible, then it would become a quiescent stem cell. Also, the rules are entirely based on the biological observations as discussed in the description of the rules. The ordering of subrules within each rule defines consistent and deterministic behavior of the model.

**Figure 3:**
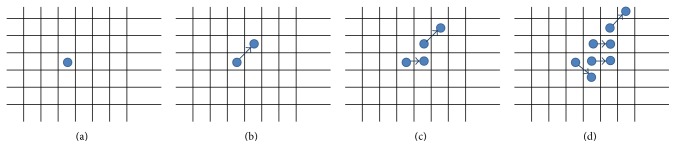
Stem cells proliferation shown progressively from images (a) to (d), with directional component moving clockwise.

Figure 3 shows how these rules when applied progressively result in one stem cell slowly occupying the entire bone marrow. The direction component initially points to the top right corner and then moves clockwise.

We show next that this proposed model of bone marrow has strong homeostatic properties, similar to Agur et al.'s model.

## 3. Homeostasis of the Bone Marrow Model

We begin by investigating the property of stem cells to expand throughout the bone marrow. The following lemma shows that any vertex in the bone marrow graph eventually gets occupied by a stem cell, given that initially there is at least one stem cell in the bone marrow graph.

**Lemma 1 .**

For any Φ, Ψ, Θ, and Δ, if there exist two vertices *u*, *v* ∈ *V* such that, at some time *t*, the vertex *v* is not occupied by a stem cell and the vertex *u* is occupied by a stem cell, then there exists a bound on the number of time steps, *s* > 0, such that the vertex *v* will be occupied by a stem cell at the most by *s* + *t* time steps.

**Proof**

From Rule (1), we conclude that if *u* and *v* are neighbors, then *u* remains a stem cell as long as *v* is not a stem cell. The vertex *v* itself turns into a stem cell in no more than Φ + *μ*Ψ time steps. This is the maximum time required which includes the time required for cell at vertex *v* to migrate to the blood stream (in case it was a differentiated cell), turn into an empty space, and, as it is a neighbor of a stem cell, become a stem cell after maximum *μ*Ψ time steps. Hence, we can use induction on the distance *ρ*(*u*, *v*) to obtain a bound on the time that is needed for *v* to turn into a stem cell:

(5) $$\begin{array}{c} s\leq \Phi +\rho \left(\;u,v\;\right)\mu \Psi . \end{array}$$

We note that as all the new stem cells have renewal number as zero, the new stem cells that are getting populated nearer to vertex *v* would not have renewal number as Δ.

The proof above conveys that the distance *ρ*_*U*(*t*)_(*v*) between a vertex *v*, which is not occupied by a stem cell at time *t*, and the subset *U*(*t*)⊆*V* of vertices which includes a stem cell vertex at time *t* is a nonincreasing function. Furthermore, there exists *s* ≤ Φ + *ρ*_*U*(*t*)_(*v*)*μ*Ψ such that *ρ*_*U*(*t*+*s*)_(*v*) = 0.

We now show that, for any time instant *r* ≥ *t* + *s*, *ρ*_*U*(*r*)_(*v*) ≤ *Mη* in any two consecutive time slots. This means that from the time *t* + *s* onwards there is always a stem cell not farther than distance *Mη* from *v* in any two consecutive time slots. As mentioned earlier, the parameter *η* denotes the distance multiple for a transitive cell to convert back to a stem cell.

**Lemma 2 .**

Suppose that a vertex *v* becomes a stem cell at time *t*_0_; then, for every *t* ≥ *t*_0_, there is a vertex *u* ∈ *B*(*v*, *Mη*) which is occupied by a stem cell at time *t* or *t* + 1.

**Proof**

A necessary condition for the production of a stem cell at a vertex *v* at time *t*_0_ is that there is a stem cell *v*′ in the near neighborhood of *v* that has reached maturity; that is, ∃*v*′ ∈ *N*(*v*), *x* ^*t*_0_−1^(*v*′) = (*S*, *∗*, Ψ, *∗*). Now, the cell at vertex *v* remains a stem cell until the last four conditions of Rule (1) hold. Therefore, if the stem cell at vertex *v* becomes a transitive cell at time *t*_1_ > *t*_0_, either it still has a stem cell neighbor at *t*_1_ or all of its neighbors become transitive cells simultaneously with *v*. If it is the first scenario, then the lemma holds. The second scenario can happen only if all the stem cells have their internal counters synchronized and reach the threshold Ψ simultaneously at *t*_1_. In such a case, either there is a stem cell in the near neighborhood of size *Mη* or vertex *v* will again convert from a transitive cell to a stem cell at time *t*_1_ + 1 as all its near neighbors are not stem cells. Thus, if *v* is not a stem cell, there is a stem cell in *B*(*v*, *Mη*) at time *t*_1_ or *t*_1_ + 1. Applying Lemma 1 ensures that until the next time the vertex *v* is occupied by a stem cell, the distance from *v* to the closest stem cell will not exceed *Mη* in any two given consecutive time instants.

A direct conclusion from Lemma 2 is the estimation for the density of stem cells in bounded vicinity. We state the same in the following lemma for graphs with bounded degree or fixed number of directions. The bone marrow can be described as a graph of bounded degree with each vertex connected only to its adjacent vertices.

We need two more notations: If the graph *G* has the property that there exists *ω* such that |*N*(*v*)| ≤ *ω*, ∀ *v* ∈ *V*, we say that *G* has* bounded degree* and write deg(*G*) ≤ *ω*. In the regular graph that we describe here, |*N*(*v*)| = *μ*; that is, every vertex is connected to its neighboring vertex in all directions (Figure 1).

The* density* of stem cells in a given finite subset of vertices *U* ⊂ *V* at time step *t* is the ratio of the number of stem cells *S* in *U* to the total number of vertices in *U* at *t*. It is denoted by *δ*_*t*_(*U*).

**Lemma 3 .**

Let *G* be a graph of bounded degree. Suppose that at some time *t*_0_ a vertex *v* is occupied by a stem cell; then, for every ball *B* = *B*(*v*, *Mη*) ⊂ *G*, lim_*t*→*∞*_ *δ*_*t*_(*B*)≥(*μ* − 1)/(*μ* ^*Mη*^ − 1).

**Proof**

By Lemmas 1 and 2, any ball of radius *Mη* admits a stem cell from a certain moment for any two consecutive time slots. Such a ball contains vertices less than or equal to (*ω* ^*Mη*^ − 1)/(*ω* − 1). As *ω* = *μ* in our graph, hence, lim_*t*→*∞*_ *δ*_*t*_(*B*)≥(*μ* − 1)/(*μ* ^*Mη*^ − 1).

**Claim 1 .**

In essence, Lemmas 1, 2, and 3 show that not only is it true that one stem cell is sufficient to bring back the bone marrow system homeostasis, but also it is true that the bone marrow has a built-in mechanism guaranteeing that stem cells do not become too scattered. Every ball of radius *Mη* is occupied by at least one stem cell at any two consecutive time steps from the moment it was occupied by a first stem cell. Also, the density of such a ball in our proposed model is at least (*μ* − 1)/(*μ* ^*Mη*^ − 1). Our claim is ratified by Parmar et al. in [19].

In the next section, we show that the proposed model has steady production of differentiated cells.

## 4. Steady Production of Differentiated Cells

In this section, we show that the system generates enough mature differentiated blood cells. Before proving the same, we mention some observations:

- When a transitive cell is created and if it has a stem cell in its near neighborhood, then it would always proceed to create a differentiated cell. This stem cell will remain a stem cell at least till the time the transitive cell becomes a differentiated cell, the differentiated cell becomes an empty space, and the empty space is occupied by another stem cell.
- If the bone marrow graph is to be completely filled with stem cells, then every stem cell should divide into two stem cells and any stem cell should neither convert to a transitive cell nor die. If any stem cell converts to a transitive cell, then the condition described above will ensure that it becomes a differentiated cell.

An extremely rare situation can occur, when the bone marrow system contains only stem cells at time *t* and the internal counters of all stem cells are synchronized. In such a case, all the stem cells will convert to transitive cells on or before *t* + Ψ. At the next time instant, all these transitive cells will convert back to stem cells, as there will not be a single stem cell in their near neighborhood. This system would not produce any differentiated cells but will also not die out. We can call such a state as a* resonant state* as the cells will resonate between stems cells and transitive cells without producing any differentiated cells. The resonant state is an artifact of the model. Later, in this section, we show that the possibility that the model will be in a resonant state is extremely rare.

**Lemma 4 .**

Suppose that a vertex *v* ∈ *V* is occupied by either a stem cell or a transitive cell at time *t*. Then, either *v* or one of its near neighbors in *B*(*v*, *Mη*) will be occupied by a differentiated cell within (*μ* + 1)Ψ + *M*Θ + 1 iterations unless the system is in a resonant state.

**Proof**

Assume that at vertex *v* there is a stem cell that has no differentiated neighbors. If there is a differentiated neighbor, then the above lemma trivially holds. *N*(*v*) will consist only of stem cells for at most *μ*Ψ time steps. Then, vertex *v* or one of its neighbors will convert to a transitive cell after Ψ time steps. Such a transitive cell will always have a stem cell in its near neighborhood. Then, after *M* generations of a transitive cell, it would convert to a differentiated cell, that is, after *M*Θ time steps.

Now, let us assume that at vertex *v* there is a transitive cell and *v* has a stem cell in its near neighborhood *B*(*v*, *Mη*); then, after (*M* − 1)Θ time steps, *v* enters the *M*th generation and in next Θ time steps it becomes a differentiated cell. If *v* is a transitive cell and if *v* does not have any stem cell in its near neighborhood that can be at a maximum distance of *Mη* from *v*, it becomes a stem cell in the next time instant and the argument above follows.

Thus, except in the case of a resonant state, there is a differentiated cell generated within every (*μ* + 1)Ψ + *M*Θ + 1 time steps in *B*(*v*, *Mη*).

**Claim 2 .**

Lemma 4 shows that, in case of severe perturbations, a transitive cell will convert back to a stem cell. This stem cell would potentially enable the bone marrow system to begin production of differentiated cells.

Note that, in this model, one cannot guarantee that a particular stem cell will eventually be converted to a differentiated cell. The lemma above does guarantee that in the close vicinity of any stem cell some cell differentiates within a fixed bounded time interval unless the system is not in a resonant state. An immediate consequence of this is a lower bound on the supply of differentiated cells to the blood stream.

**Lemma 5 .**

Suppose that, at some time *t*_0_, a vertex *v* is occupied by a stem cell; then, every ball of radius 2*Mη* eventually supplies at least one mature differentiated cell within (*μ* + 1)Ψ + *M*Θ + 1 + Φ time steps unless the system is in a resonant state.

**Proof**

By Lemma 3, every ball of radius *Mη* admits a stem cell from a certain moment onwards in any two consecutive time instants. Lemma 4 states that either this stem cell or one of its near neighbors (and so we argue about balls of radius 2*Mη*) converts to a differentiated cell within (*μ* + 1)Ψ + *M*Θ + 1 time steps and migrates from the bone marrow as mature differentiated cell after Φ additional time steps. Thus, every ball of radius 2*Mη* eventually supplies at least one mature cell within (*μ* + 1)Ψ + *M*Θ + 1 + Φ time steps.

We will now try to reason out the fact that the chances of occurrence of a resonant state are rare. A resonant state can occur for a block of holding capacity of 2^*μ*^ cells if it is occupied completely by stem cells starting from a single stem cell in *μ*Ψ time steps. The physical occupancy of stem cells in a given block depends largely on the initial stem cell population and the round-robin way of choosing the directions. If the manner in which the round-robin arrangement of directions is clockwise or counterclockwise, then the resonant state would not occur if starting with a single cell in two-dimensional space, as 2^*μ*^/*μ* ^2^ is greater than 1 when *μ* is greater than 4. For example, with *μ* = 8 in a two-dimensional space, 2^8^ = 512; thus, in 8Ψ time steps, 512 cells would be generated from a single stem cell, but the ball of radius 8 from the vertex *v* can hold at a maximum only (8 + 1 + 8)^2^ = 289 number of cells. Thus, there will be at least one stem cell, which would have all its neighbors as stem cells within 8Ψ time steps, and hence it would get converted to a transitive cell. In our model, since the directional component is assumed to be clockwise, the resonant state will never occur. The resonant state may occur rarely in cases when the directional component is chosen arbitrarily. The possibility of reaching a resonant state drops further after considering the coordination of a similar event in neighboring blocks, required for the entire bone marrow system to be in the resonant state.

It is observed that several stem cells do remain in quiescent state [8]. Quiescent state is defined as the state of a stem cell such that even after the cycling phase has reached the threshold Ψ, it remains a stem cell instead of converting to a transitive cell.

**Lemma 6 .**

For every Φ, Ψ, and Θ and with Δ sufficiently large, several stem cells remain in quiescent state.

**Proof**

The above analysis shows that if the number of renewals Δ, allowed for stem cells, is sufficiently large, then the stem cells would not die for a sufficiently long time; instead, they would proliferate and fill up the entire surrounding space. Thus, in case when a stem cell *s* turns to a transitive cell, the stem cells in the near neighborhood of *s*, that is, *N*(*s*), would neither be able to proliferate nor convert to a transitive cell, as they are no longer surrounded by stem cells from all directions. These stem cells would then enter quiescent state after Ψ time steps.

## 5. Steady States and Dying Out States

We consider the unique state of the bone marrow satisfying ∀*v* ∈ *V*, *x*(*v*) = (*E*) as the* death state* of the system. A state *x* ^*t*^, for which there exists *k* ∈ *Z* ^+^ such that *x* ^*t*+*k*^ is the death state, will be called a* dying out state*.

**Lemma 7 .**

The dying out states of the bone marrow are only those consisting of no stem cells or no transitive cells or the states which reach ∀*v* ∈ *V*, *x*(*v*) = (*S*, *d*, Ψ, Δ).

**Proof**

Let *x* ^*t*^ ∈ *Ω* be a state of bone marrow, which is not one of the dying out states. Let us assume that at time *t* there is a vertex *u*, which has a stem cell. If there exists a vertex *v* ∈ *V*, which is not a stem cell at time *t*, *v* becomes a stem cell by Lemma 1. So, by Lemma 2, there always exists a stem cell in *B*(*v*, *Mη*) in any two consecutive time instants after vertex *v* is occupied by a stem cell. The system does not die out. Even if *v* is a transitive cell, then it will become a stem cell if there is no stem cell in its near neighborhood. So, again, the system does not die out.

Consider an extremely rare case where the bone marrow system is occupied by synchronized stem cells that all have reached the maturity time Ψ and that all already have had Δ proliferations; that is, ∀*v* ∈ *V*, *x*(*v*) = (*S*, *d*, Ψ, Δ). In such a scenario, all the stem cells of the bone marrow would execute first subrule of Rule (1) and would become empty spaces. The system would die out. Thus, assume that *V* admits only stem cells at time *t*. If the counters are not synchronized, they do not convert to transitive cells at the same time instant and the system does not die out. If the counters of all stem cells are synchronized and all the stem cells have not had Δ proliferations, they enter the resonant state and again the system does not die out. If the counters of all stem cells are synchronized and all the stem cells have had Δ proliferations, only then the system dies out.

We have shown that except for the rare chance of occurrence of a death state, the system never dies out. The chance of the bone marrow system being in a resonant state is also low. Thus, we have shown that the model representing the bone marrow is in the state of dynamic equilibrium that it continuously changes in its constitution and arrangement, and these changes occur at varying rates depending on the constants Φ, Ψ, Θ, and Δ. Thus, the bone marrow model can be considered as if it is in homeostasis.

If there exist states *x* ∈ *Ω* in which, for some *k* ∈ *Z* ^+^, *x* ^*t*+*k*^ = *x* ^*t*^, then all these states from *x* ^*t*^ to *x* ^*t*+*k*^ are the* steady states* of the system.

**Lemma 8 .**

For every Φ, Ψ, Θ, and Δ, the model reaches a steady state.

**Proof**

Given the fact that the number of states is finite, the model will eventually repeat states.

**Claim 3 .**

The bone marrow model displays homeostatic behavior except if it is in a death state or in a resonant state.

## 6. Discussion

In this paper, we have proposed a biologically consistent deterministic model of bone marrow by extending the model proposed by Agur et al. The proposed model not only retains homeostatic properties of Agur et al.'s model but also adds the ability to recover from severe perturbations of the bone marrow by adding rules that can convert a transitive cell back to a stem cell and bring back the system homeostasis. The model demonstrates consistency to assume a certain apoptosis rate in the stem cell population.

The main properties of our model are achieved from the feedback demand of Rule (1); namely, a stem cell does not convert to a transitive cell unless its immediate microenvironment is saturated with stem cells. The feedback demand in Rule (2) is also significant in the sense that a transitive cell can convert back to a stem cell in cases of severe perturbations resulting in loss of several stem cells. We obtain the results that stem cells are eventually dense (Lemmas 2 and 3) and that, except for the cases when there is no stem cell or no transitive cell or bone marrow state having all synchronized stem cells having had Δ renewals, the bone marrow system never dies out (Lemma 7). Even though our extension of Agur et al.'s model is relatively simple, the properties that emerge are general and hold for more complex descriptions. It is a step ahead in the direction to model the immensely complex bone marrow system.

Our extension of Agur et al.'s model removes the drawbacks associated with it. To summarize, note the following:

(1) The model demonstrates that a single HSC is sufficient to bring back bone marrow homeostasis. Osawa et al. [13] have also shown that injection of a single HSC resulted in a long term reconstitution of the hematopoietic system.
(2) The model demonstrates consistency with apoptosis observed in the stem cell population. As pointed out by Roeder and Radtke [1], stem cells accumulate DNA damage with each cell division. DNA damage that exceeds a certain level induces apoptosis or cell cycle arrest [15].
(3) In the model, a stem cell division is explicitly represented. A stem cell's internal counter comes back to its initial state after division; that is, it becomes a true daughter stem cell. Thus, a stem cell divides into two identical daughter stem cells. Yamamoto et al. [20] demonstrated symmetric self-renewal division of HSCs as an actual event.
(4) One stem cell divides into a single empty space. For another division, the stem cell has to wait for the internal counter to again reach threshold Ψ.
(5) Our model shows that a stem cell can enter and exit a quiescence and a proliferative state. Wilson et al. [21] have discussed that the HSCs regularly enter and exit cell cycle. After reestablishment of homeostasis, HSCs return to quiescence, suggesting that HSCs are not permanently entering the cell cycle but reversibly switch from quiescence to self-renewal under certain conditions.
(6) Transitive cells that have limited ability to convert back to stem cells are represented in our model. Their ability to convert back to a stem cell diminishes with subsequent generations. Yin et al. [14] have shown that the intermediate cells can reconstitute hematopoietic stem cells. They also show that these dedifferentiated HSCs can reconstitute entire hematopoietic function.
(7) Our model has empty spaces but they no longer need any counters.
(8) The results hold in two-dimensional or three-dimensional model of space.
(9) The homeostatic model allows flexibility in choosing model parameters that permits several different simulations to be carried out in silico (Claim 3).

Our model overcomes the drawbacks of Agur et al.'s model. It also does not require explicit message passing between cells and the controlling microenvironment, as required by the model of D'Inverno and Saunders [7]. Compared with the other proposed models [10, 11, 16], our model has transitive cells with several generations; homeostasis prevails for a larger range of parameter values; and there is no fixed turnaround rate between the proliferative state and the quiescent state of the stem cells. Hence, it is much closer to biologically observed behavior of bone marrow than the earlier reported deterministic models. The properties of the proposed model proved in this paper are also observed in the agent-based simulations that we have carried out. These simulations also demonstrate that, as predicted, large fractions of stem cells do remain in quiescent state [8].

There are other advantages of our model. It can be modified to qualitatively study some of the blood related diseases. For example, anemia is usually defined as decrease in the number of red blood cells (RBCs). Aplastic anemia is a type of anemia that is caused by the disturbance of proliferation and differentiation of hematopoietic stem cells. Aplastic anemia can be caused by exposure to certain chemicals, drugs, radiation, infection, immune disease, and heredity. Yet in about half the cases, the cause is unknown [22]. In essence, if the maturing and dying differentiated cells are not replaced by newer differentiated cells at the same rate, then it may lead to aplastic anemia. A stem cell converts to a transitive cell in Ψ time steps if it is surrounded by stem cells in all directions. Each of these conversions will potentially create a differentiated cell. These differentiated cells would mature and die in Φ time steps. Thus, the number of live differentiated cells would largely depend upon the ratio (Φ/Ψ). Thus, if the ratio (Φ/Ψ) is very low then it may cause aplastic anemia. This is one example of a possible extension of the model defined in this paper.

There are several other options of bringing the proposed model even more close to biologically observed complexity. We would like to increase the scale of simulation of the bone marrow system so that it can hold 10^8^ to 10^12^ number of cells [23]. We would also like to perform simulations in three-dimensional space. In future, we would also like to model leukemia as has been done in other models [16].

## Figures and Tables

**Figure 4:**
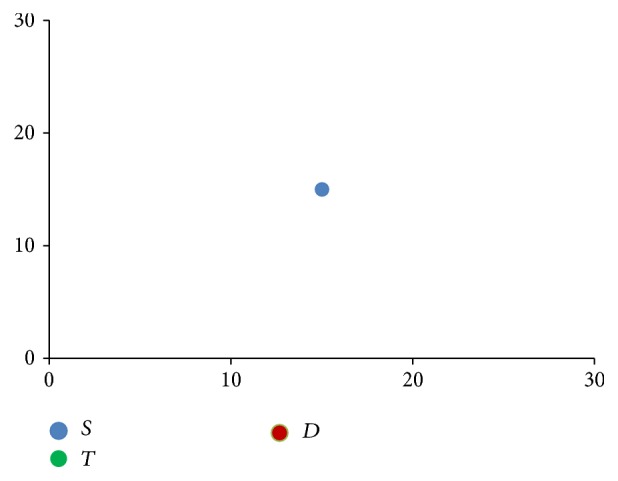
Initial state with a single stem cell.

**Figure 5 fig5:**
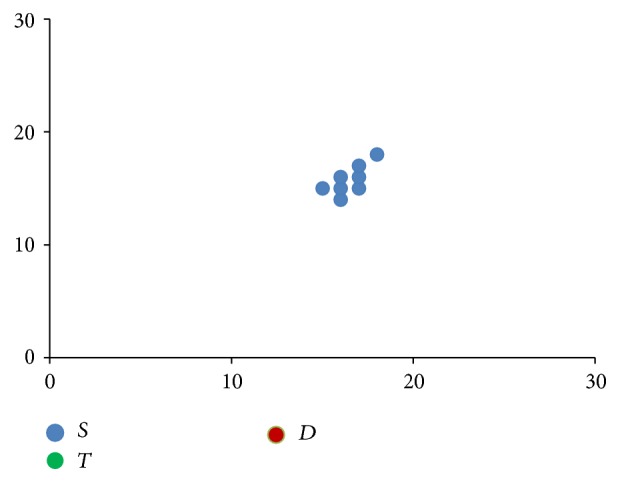
After 20 time steps, 0% stem cells quiescent.

**Figure 6 fig6:**
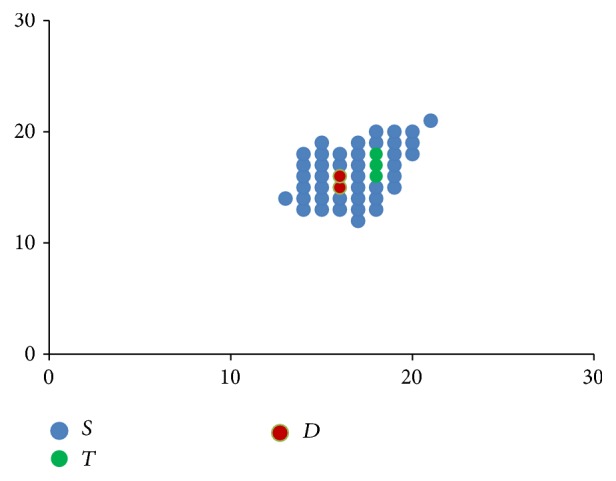
After 50 time steps, 12.2% stem cells quiescent.

**Figure 7 fig7:**
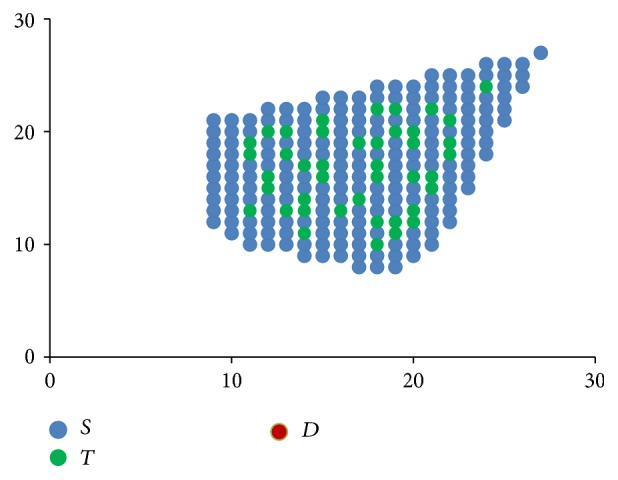
After 100 time steps, 43.32% stem cells quiescent.

**Figure 8 fig8:**
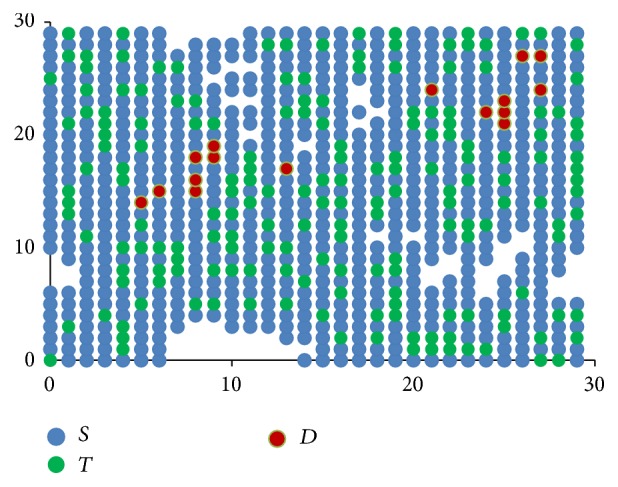
After 200 time steps, 60.56% stem cells quiescent.

**Figure 9:**
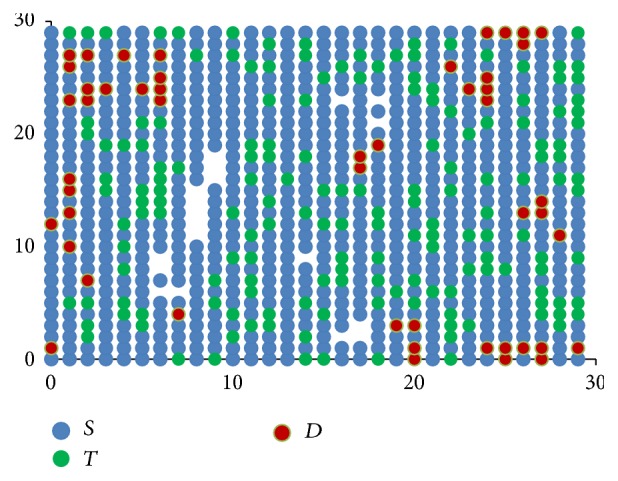
After 500 time steps, 59.22% stem cells quiescent.

**Figure 10:**
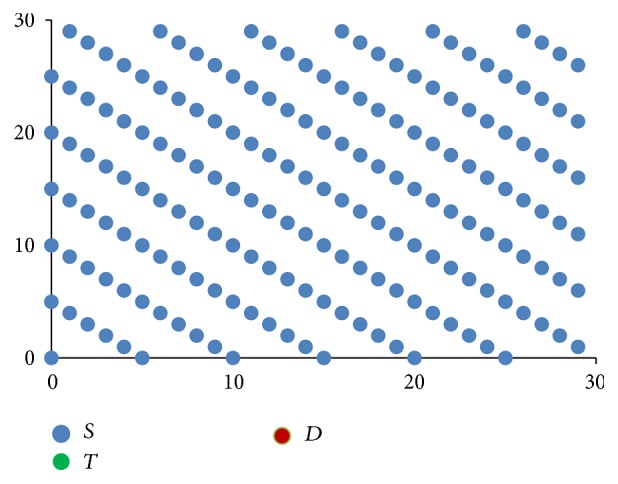
Starting with 20% evenly distributed stem cells.

**Figure 11:**
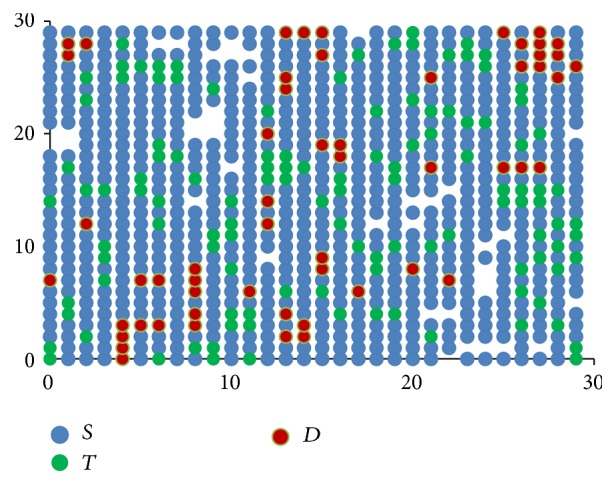
After 500 time steps, 58.95% stem cells quiescent.

**Figure 12:**
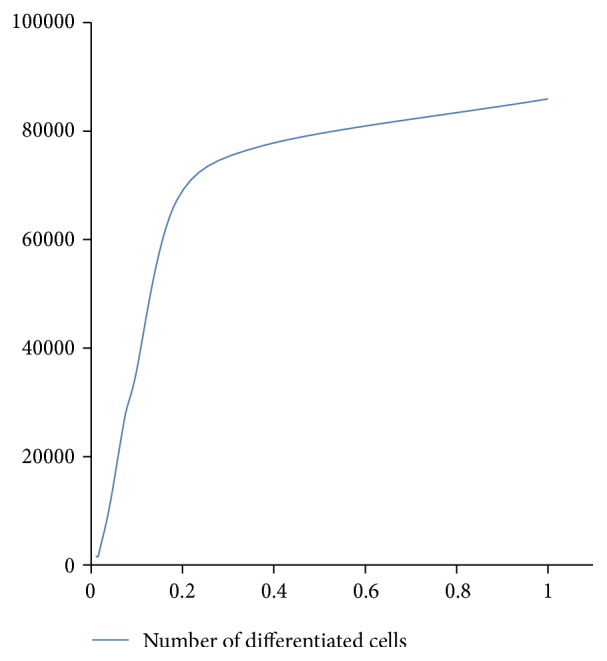
Number of differentiated cells (*y*-axis) against (Φ/Ψ) ratio (*x*-axis) over 1000 time steps.

## Appendix

### Results of the Agent-Based Simulation

An agent-based simulation was developed for the bone marrow system proposed in this paper. We have implemented the program in the C programming language and it is made available at a publicly available website: https://sites.google.com/site/deterministicstemcellmodel/.

The results given below are for a two-dimensional grid of size 30 × 30 for the following constants (in figures, blue is used for stem cells, green is used for transitive cells, and maroon is used for differentiated cells):

Δ = 100, number of maximum renewals for a stem cell.
Φ = 2, constant for differentiated cells maturity.
Ψ = 8, constant for stem cells maturity.
Θ = 3, constant for transitive cells maturity.
*η* = 1, constant for distance measure for near neighborhood.
*μ* = 8, number of directions in a two-dimensional space.
*M* = 3, number of generations for a transitive cell.

We have considered the two-dimensional space as circular; that is, the last column is neighbor to the first column and the last row is neighbor to the first row. The directional component initially points to the top right corner and then moves in a clockwise manner.

Simulation 1 (starting with a single stem cell in the center of the grid (Figures 4 [Fig fig5] [Fig fig6] [Fig fig7] [Fig fig8]–9)). This simulation captures the emergent phenomenon of different cells occupying the entire bone marrow starting with a single stem cell. The simulation demonstrates the ability of the bone marrow to recover from severe perturbations. It shows the production of differentiated cells. It also shows that some stem cells are in quiescent state.

Simulation 2 (starting with 20% evenly distributed stem cells (Figures 10 and 11)). This simulation demonstrates the emergent properties for a normal bone marrow. In a normal bone marrow, the stem cells would be distributed across the tissue. Hence, this simulation starts with evenly distributed stem cells in the bone marrow. Eventually, different cells, including differentiated cells, occupy the entire bone marrow.

Simulation 3 (number of differentiated cells depend on the (Φ/Ψ) ratio (Figure 12)). The figure shows a plot of (Φ/Ψ) ratio against the number of live differentiated cells over 1000 time steps. This graph is plotted by varying values of Ψ while keeping all the other constants unchanged. It can be observed that if the (Φ/Ψ) ratio is low, then the number of live differentiated cells is also low, and that may result in aplastic anemia.
